# HLA Associations in pediatric autoimmune liver diseases: Current state and future research initiatives

**DOI:** 10.3389/fimmu.2022.1019339

**Published:** 2022-09-29

**Authors:** Cara L. Mack

**Affiliations:** Department of Pediatrics, Division of Pediatric Gastroenterology, Hepatology & Nutrition Medical College of Wisconsin and Children’s Wisconsin, Milwaukee, WI, United States

**Keywords:** autoimmune hepatitis, autoimmune sclerosing cholangitis, major histocompatibility complex (MHC), primary sclerosing cholangitis, T cell activation

## Abstract

The strongest genetic association with autoimmunity is within chromosome 6p21, where the human leukocyte antigen (HLA) complex resides. This review will focus on the HLA associations within pediatric autoimmune hepatitis, autoimmune sclerosing cholangitis and primary sclerosing cholangitis. In general, there is considerable overlap in HLA genotypes conferring susceptibility to pediatric autoimmune liver diseases, however unique HLA associations and protective HLA genotypes exist. There are numerous areas for future research initiatives in pediatric autoimmune liver diseases and HLA associations with clinical outcomes, autoantigen discovery and novel therapeutics targeting the HLA- autoantigen- T cell pathway will be highlighted.

## Overview of the role of the human leukocyte antigen complex in autoimmunity

A unifying theory on the pathogenesis of autoimmune liver diseases includes an infectious or environmental trigger in the genetically predisposed individual that is associated with an aberrant chronic autoimmune response targeting the hepatocyte or cholangiocyte. The strongest genetic association with autoimmunity is within chromosome 6p21, where the HLA complex resides. The HLA nomenclature is specific to humans, and is inclusive of the broader terminology for other animals, namely the major histocompatibility complex (MHC). There are two classes of HLA molecules- MHC class I (HLA *A, -B, -C*) which are present on most nucleated cells and MHC class II (HLA *DP, -DQ, -DR*) that are present predominantly on professional antigen presenting cells (APCs) (dendritic cells, B cells, macrophages). MHC class II molecules are a heterodimer of an alpha (α) and beta (β) chain. In the example of HLA *DR*, the α- chain is encoded by the HLA *DRA* locus and the β- chain is coded by either HLA *DRB1, ;-DRB3, -DRB4* or *-DRB5* loci. Furthermore, phenotyping of the HLA molecule of interest may be denoted numerically, reflecting a specific allele within the locus (i.e. HLA DRB1*0301) (1).

The T cell activation that predominates in many autoimmune diseases and contributes to disease pathology is dependent on the APC presenting the antigenic peptide complexed to MHC to the T cell receptor (“signal 1”), as well as the APC providing co-stimulation to the T cell (“signal 2”). Certain HLA types are associated with outcomes in specific autoimmune diseases such as type 1 diabetes (2) and celiac disease (3), suggesting a direct link of HLA with disease pathogenesis. This review will focus on the HLA associations of autoimmune liver diseases (AILD) in children, namely autoimmune hepatitis (AIH), primary sclerosing cholangitis (PSC) and autoimmune sclerosing cholangitis (ASC; also known as AIH-PSC overlap syndrome). ASC is defined as meeting criteria for both AIH and having radiographic and/or histologic evidence of PSC (4). Unique HLA associations reported in distinct geographical areas and pediatric populations will be highlighted.

## HLA associations in pediatric AILD

### AIH and ASC

The majority of reports of HLA associations with AILD are from adults and include HLA class I (A*01, B*08) and HLA class II alleles (DRB1*03, -04, -07 and/or -13) *(* 5). Children with AILD are unique compared to their adult counterparts based on higher incidences of type 2 AIH [anti-liver kidney microsomal type 1 (LKM1) and/or anti-liver cytosol type 1 (LC1) antibody positivity] and ASC (6). The largest cohort of children with AIH or ASC that have had detailed HLA association analyses are from King’s College Hospital in London, England (7). A current report from this institution entailed outcome analyses of 236 European children with type 1 AIH (ANA and/or anti-actin positivity), type 2 AIH or ASC. With respect to MHC class I alleles, significantly higher frequencies of HLA A*01 occurred in type 1 AIH and HLA B*8 in all groups compared to healthy controls. MHC class II alleles that were more predominant in type 1 AIH included HLA DRB1*03 and HLA DQ*02. The presence of homozygosity for HLA DRB1*03 conferred the highest risk for AIH and ASC compared to healthy controls. Linking MHC class I and II expression, the HLA A*01-B*8-DRB1*03 phenotype frequency was significantly higher in all groups compared to healthy controls. ASC susceptibility was further delineated based on having both HLA B*8 and homozygous DRB1*03 phenotype or being HLA DRB1*13 positive/DRB1*03 negative. Unique to type 2 AIH patients was a significantly higher frequency of HLA DRB1*07 compared to all other groups.

HLA associations with clinical presentation and outcomes in the King’s College cohort revealed that baseline AST levels were highest in those expressing HLA DRB1*03 and DRB1*07 (type 1 and 2 AIH susceptibilities, respectively) compared to HLA DRB1*13 (ASC susceptibility), while those with HLA DRB1*13 had higher baseline levels of alkaline phosphatase and GGT. This provides indirect evidence that HLA genotypes contribute to disease pathogenesis, as the marker of hepatocytic injury (AST) was highest in AIH-associated HLA genotypes and cholestatic enzymes reflecting ASC were highest in the HLA DRB1*13 cohort. Further evidence of HLA contribution to disease is based on the findings that more significant histological inflammation and fibrosis occurred in those children with either HLA DRB1*03 (homozygous) or DRB1*13 compared to other HLA DR genotypes.

With regard to outcomes, patients heterozygous for DRB1*03 had a shorter time to remission, a higher “good responder” status and a lower frequency of end stage liver disease compared to those with DRB1*07 or DRB1*13. Despite the fact that DRB1*03 was associated with better outcomes, there was no HLA risk allele associated with need for liver transplant. Since liver transplant occurs in only 15-20% of children with AIH (8), a much larger cohort of patients would be needed to determine if a given HLA genotype is an independent risk factor for need for liver transplant.

Junge et al. (9) performed targeted high-resolution genotyping of HLA *DRB1* in 67 children with AILD from Germany. The frequency of HLA DRB1*0301 (heterozygous or homozygous) was significantly higher in type 1 AIH and ASC compared to healthy controls. However, HLA DRB1*1301 frequency was significantly higher only in the type 1 AIH, unlike the King’s College experience where it predominated in ASC. The AIH group from Germany did not undergo routine screening for ASC and therefore it is unclear if those with HLA DRB1*1301 were actually AIH versus ASC. Finally, type 2 AIH had a high frequency of HLA DRB1*0701 compared to type 1 AIH, comparable to the King’s College report.

**Figure 1:**
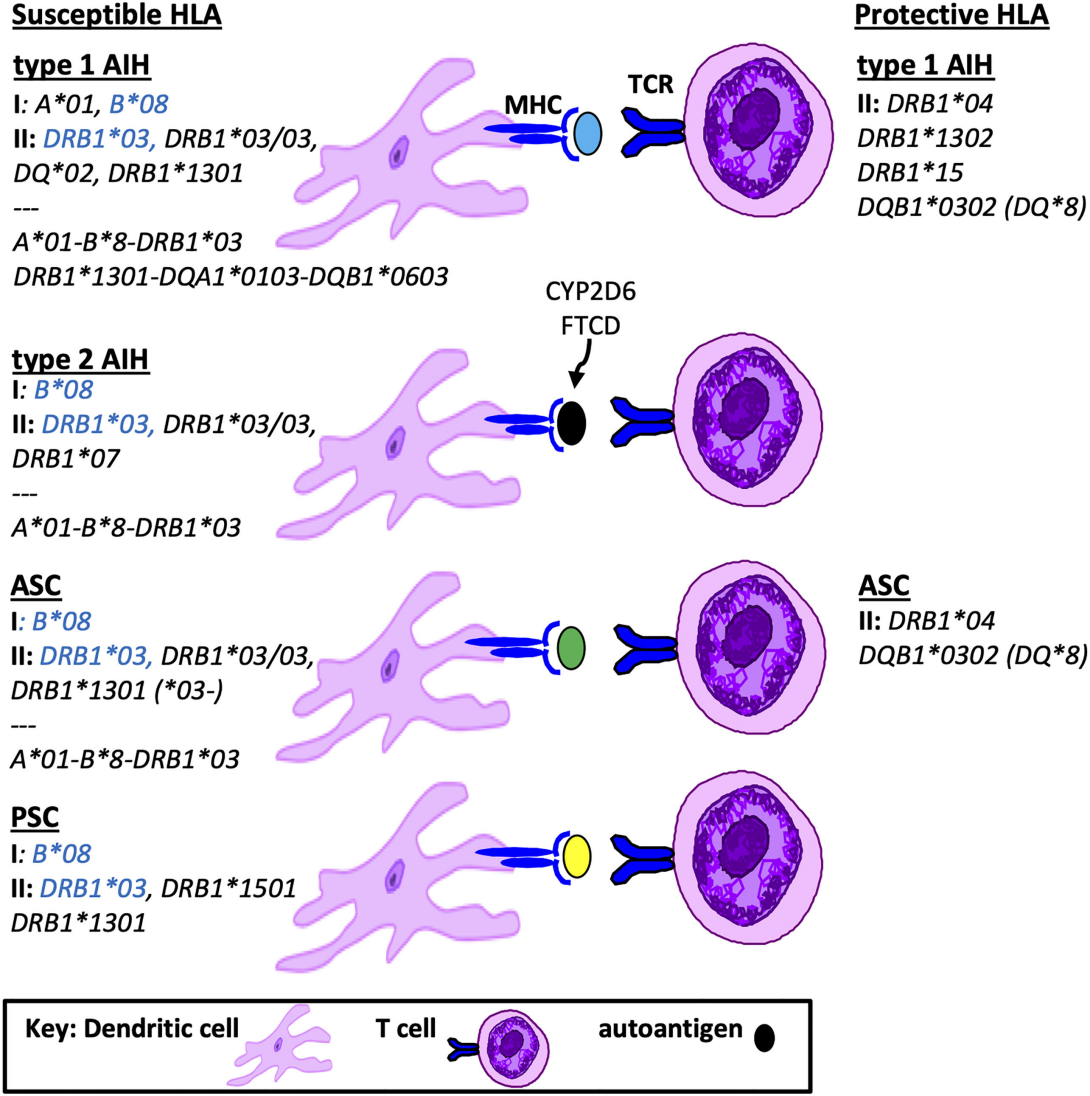
Susceptible and Protective HLA genotypes in Pediatric Autoimmune Liver Diseases. Shown is a summary of HLA genotypes associated with pathogenesis or protection from autoimmune liver diseases in children, including MHC class I, class II and grouped haplotypes based on linkage disequilibrium. Highlighted in blue are susceptibility HLA genotypes found in all pediatric AILD.

In South America, a recent study from Brazil performed targeted genotyping of HLA *DRB1* in 43 children with either type 1 AIH or ASC (10). When compared to healthy controls, the frequency of HLA DRB1*03 and HLA DRB1*13 were significantly higher in the both groups compared to controls. Furthermore, the presence of HLA DRB1*13 conferred a 3-fold increase risk for ASC. A landmark study from Argentina in 1999 described MHC class II genotyping in 122 children with type 1 AIH (11). HLA DRB1*1301 was identified as the primary susceptibility allele for AIH. However, there was no description of screening for ASC in this population and therefore accurate differentiation of the HLA DRB1*1301 association with AIH versus ASC was not possible. The exclusion of children who were HLA DRB1*1301 positive uncovered a second association of AIH with HLA DRB1*0301. Two other HLA genes that map close to HLA *DRB1* are HLA *DQA1* and *DQB1*. Both HLA DQA1*0103 and DQB1*0603 were found in strong linkage disequilibrium with DRB1*1301 in AIH; thus the risk haplotype was defined as HLA DRB1*1301-DQA1*0103-DQB1*0603. A summary of HLA susceptibility genes in AIH and ASC are provided in **Figure 1**.

### PSC

In contrast to the robust HLA mapping in pediatric AIH, there is a paucity of information on HLA associations in children with PSC. In adults, some of the HLA associations with PSC include HLA B*07, B*08, and DRB1*1301 (12, 13). One of the first reports of HLA associations in pediatric PSC by Wilschanski et al. (14) from Canada showed that the frequency of HLA B*8 and DRB1*1501 in PSC was significantly greater than in controls. Brazil reported on 27 children and 36 adults with PSC and found that, similar to adults, DRB1*1301 inferred susceptibility in children (15). Ylinen et al. (16) performed HLA genotyping in 19 children with AILD from Finland, including 7 with PSC, compared to 19,807 controls. Higher frequencies of HLA B*08, DRB1*03 and DRB1*13 occurred in AILD, however there was no difference in these frequencies when comparing AIH to PSC. The German study by Junge et al. detailed above for AIH and ASC also included 20 children with PSC (9). Significantly higher frequencies of HLA DRB1*0301 and DRB1*1301 were found in PSC compared to controls.

## HLA genotypes that are protective from AILD

HLA genotypes that are lower in frequency in a disease state compared to the general population implies that those genotypes may actually protect from developing an autoimmune disease. In the King’s College study, protective HLA associations in AIH were inferred based on significantly lower frequencies of HLA DRB1*15 in children with type 1 AIH (7). Similarly, in adults with AIH, HLA DRB1*1501 was associated with protection from disease (17). The peptide binding pocket of HLA DRB1*1501 differs from the susceptibility HLA DRB1*0301 by one amino acid at position 71 (alanine (17) versus lysine (18)), suggesting that this is a key HLA position associated with the risk of developing AIH.

The pediatric King’s College study also identified DRB1*04 and DQB1*0302 (DQ*8) as protective in type 1 AIH and ASC compared to healthy controls (7). With regard to HLA DRB1*04, the aforementioned pediatric Finnish report likewise showed no HLA DRB1*04 positivity in those with either AIH, ASC or PSC (16). In contrast, HLA DRB1*04 is considered a disease susceptibility HLA genotype in adults with AIH (5). The DRB1*04 risk allele in adult AIH is associated with a later age of onset of AIH (19) and likely represents a novel subtype of AIH not found in children.

Another HLA genotype that was found to be protective for AIH in the Argentinian pediatric cohort was HLA DRB1*1302 ( 11 *)*. Interestingly, HLA DRB1*1302 differs by only one amino acid (valine) from the susceptibility HLA DRB1*1301 (glycine) in position 86 of the peptide binding pocket, suggesting that this position is key to antigen presentation. With regard to protective HLA genotypes in PSC, there is no data in children. However in adults with PSC the haplotype HLA DRB1*04-DQB1*03 represents the most consistent protective HLA association (13). A summary of susceptible and protective HLA associations in children is provided in **Figure 1**.

## Future research initiatives focused on HLA associations in pediatric AILD

In general there is considerable overlap in HLA associations between pediatric AIH, ASC and PSC, with all three AILD types reporting associations with either HLA B*08, DRB1*03 and/or DRB1*13. Type 2 AIH has a unique HLA DRB1*0701 predominance and PSC has an additional HLA DRB1*1501 susceptibility. The accuracy of these associations as it relates to AIH versus ASC is limited in the cohorts that were not systematically screened for ASC. Protective HLA genotypes in children include HLA DRB1*04 (AIH, ASC, PSC), DRB1*1302 (type 1 AIH), DRB1*15 (type 1 AIH*)* and DQB1*0302 (AIH, ASC). In comparison to adult AILD HLA associations, there are both redundant and unique susceptibility and protective HLA genotype associations in children.

In adults with AIH, racial and ethnic differences in outcomes have been reported (20), however little is known about these racial/ethnic groups and HLA associations in pediatric AILD. In addition, in many countries such as North America and Asia, rigorous analyses of HLA associations in pediatric AILD have not performed. Only the recent King’s College report analyzed clinical presentation and outcome correlations with HLA genotypes. Herein lies the first future research initiative, whereby HLA association studies worldwide should include sub-analyses based on race/ethnicity and outcomes analyses in order to provide evidence of HLA-associations with prognosis (**Table 1**).

**Table 1:** Future Research Initiatives in Pediatric AILD.

Worldwide Epidemiology & Health Outcomes Research	
AIH, ASC	Determine HLA associations with clinical outcomes (e.g. disease severity at presentation, need for liver transplant, etc.)
PSC	Define HLA associations and determine associations with clinical outcomes
AIH, ASC, PSC	Determine HLA associations with race/ethnicity
*Pediatric AILD Pathogenesis Research*	
AIH (type 1), ASC, PSC	Determine autoantigen(s) involved in MHC- restricted T cell activation
AIH (type 1), ASC, PSC	Create novel mouse models of disease to study mechanisms of MHC- restricted T cell activation
AIH (type 1), ASC, PSC	Utilize the HLA3D toolkit for autoantigen discovery
*Novel therapies targeting the MHC- peptide- T cell pathway*	
AIH, ASC, PSC	Implement nanoparticle technology to enhance immunoregulation of autoimmunity

The second understudied area relates to pediatric AILD pathogenesis. There is a paucity of data on the autoantigens that are being presented to the T cell in an MHC- restricted fashion. Only in type 2 AIH have the autoantigens been discovered, specifically cytochrome P450-2D6 (CYP2D6) (21, 22) and formiminotransferase-cyclodeaminase (FTCD) (23). FTCD is the autoantigen recognized by anti-LC1 autoantibodies that are found in a subset of type 2 AIH patients. CYP2D6 is the antigenic target of the LKM1 autoantibody, which is the hallmark autoantibody in type 2 AIH. Longhi et al. utilized MHC-peptide tetramer technology and found that HLA A*0201 CYP2D6-specific CD8 T cells in children with type 2 AIH were highest in frequency at the time of diagnosis, decreased in response to immunosuppressive therapy and correlated with hepatocyte injury (22). Recently, a new mouse model of type 2 AIH was described, whereby BALB/c mice were immunized with CYP2D6 in complete Freund’s adjuvant (to activate innate immunity) (24). These mice produced high levels of anti-LKM antibodies and CYP2D6-specific T cells and liver histology mimicked the human disease. This new model will be instrumental in discovering novel immune pathways associated with hepatocyte injury and fibrosis in type 2 AIH. Recently, a new technique for MHC-restricted autoantigen detection known as “HLA3D” was reported (25). HLA3D is a comprehensive platform that directly analyzes the interactions between HLA molecules and peptides based on the HLA conformational structure. The HLA3D toolkit integrates HLA conformational differences with antigenic peptide prediction and will likely be a powerful tool for uncovering MHC-restricted autoantigens in AILD (**Table 1**).

The third area for future research entails the discovery of therapies that specifically target the MHC- peptide (autoantigen)-T cell pathway, thus providing a powerful therapeutic approach to induce remission of AILD. Exciting work from Umeshappa et al. used nanoparticle technology to coat nanoparticles with disease associated, liver autoantigen peptides that were coupled to disease-susceptibility MHC class II molecules (26). The nanoparticles were tested in a spontaneous mouse model of primary biliary cholangitis (PBC) (the NOD.*c3c4* mouse), as well as a humanized mouse model of PBC that utilized human PBC HLA susceptibility genotypes. In both models, administration of the nanoparticle coated with the PBC autoantigen pyruvate dehydrogenase complex-E2 (PDC-E2) coupled to MHC class II resulted in protection from disease. This protection was attributed in part to the expansion of autoantigen-specific regulatory T cells that inhibited pathogenic T cell mediated liver injury. Strikingly, this response was not unique to the PBC mouse model, and similar protection of disease was demonstrated in PSC (*abcb4* ^-/-^) and AIH mouse models in response to administration of the PDC-E2-MHCII nanoparticles. The authors clearly demonstrated the ability of ubiquitous autoantigen-based-MHC nanomedicines to blunt liver autoimmunity in an organ-specific rather than disease-specific manner. It was theorized that the initial liver tissue injury in AILD has the potential to trigger activation of autoreactive T cells that recognize many additional autoantigens, thus leading to the pervasive regulatory T cell responses capable of diminishing pathology. This and other novel approaches (27) that manipulate the MHC- peptide- T cell pathway will provide powerful therapies for autoimmune liver diseases.

## Author contributions

CM researched and wrote the entire manuscript and created the figure independently.

## Conflict of interest

The author declares that the research was conducted in the absence of any commercial or financial relationships that could be construed as a potential conflict of interest.

## Publisher’s note

All claims expressed in this article are solely those of the authors and do not necessarily represent those of their affiliated organizations, or those of the publisher, the editors and the reviewers. Any product that may be evaluated in this article, or claim that may be made by its manufacturer, is not guaranteed or endorsed by the publisher.

## Abbreviations

AIH, autoimmune hepatitis; AILD, autoimmune liver disease; APC, antigen presenting cell; ASC, autoimmune sclerosing cholangitis; HLA, human leukocyte antigen; PSC, primary sclerosing cholangitis.
